# Antifungal properties of carvone and linalool against *Malassezia* species: Preliminary Screening Study

**DOI:** 10.22034/cmm.2024.345248.1547

**Published:** 2024-11-25

**Authors:** Somayeh Yazdanpanah, Aida Iraji, Solmaz Mirzamohammadi, Kamiar Zomorodian

**Affiliations:** 1 Department of Parasitology and Mycology, School of Medicine, Shiraz University of Medical Science, Shiraz, Iran; 2 Stem Cells Technology Research Center, Shiraz University of Medical Sciences, Shiraz, Iran; 3 Research Center for Traditional Medicine and History of Medicine, Department of Persian Medicine, School of Medicine, Shiraz University of Medical Sciences, Shiraz, Iran; 4 Pharmaceutical Sciences Research Center, School of Pharmacy, Shiraz University of Medical Sciences, Shiraz, Iran; 5 Basic Sciences in Infectious Diseases Research Center, School of Medicine, Shiraz University of Medical Sciences, Shiraz, Iran

**Keywords:** Antifungal agents, Carvone, Linalool, *Malassezia*, Natural products

## Abstract

**Background and Purpose:**

By harnessing the power of nature, researchers can potentially discover new therapeutic options that are safe, effective, and sustainable for the management of diseases. Recently, natural products have been extensively studied for the treatment of diseases due to their diverse chemical composition and potential therapeutic properties. Therefore, this study aimed to investigate the antifungal activity of carvone and linalool against *Malassezia* species to find alternative treatments for pityriasis versicolor.

**Materials and Methods:**

The *in vitro* antifungal activity of monoterpenes was assessed using a microdilution method, following the guidelines specified in the Clinical and Laboratory Standards Institute document M27-A3 with modifications, including the use of Christensen’s urea broth supplemented with various lipids to optimize the growth condition for *Malassezia*.

**Results:**

The minimum inhibitory concentration ranges for linalool and carvone were found to be 0.3-5.4 and 0.3-24 mg/mL, respectively. Additionally, the growth of *Malassezia* species was inhibited at concentrations of 0.001-0.003 and 0.006-0.1 mg/mL for amphotericin B and ketoconazole, respectively.

**Conclusion:**

Given the remarkable antifungal properties exhibited by linalool and carvone against *Malassezia* species, these terpene compounds have the potential to be utilized for the treatment of *Malassezia* infections, provided that additional research is conducted.

## Introduction

*Malassezia* is a genus of fungi that belongs to the Basidiomycota phylum and comprises part of the skin microbiota in humans and animals. Through molecular identification, 18 species of Malassezia have been classified until now [ 1 ]. Some Malassezia species have been associated with skin conditions, like pityriasis versicolor, folliculitis, dandruff, and seborrheic dermatitis. *Malassezia* species has also been reported in bloodstream infections, particularly in premature neonates and hospitalized patients receiving intravenous lipids [ 2 ], and may play a role in exacerbating psoriasis and atopic dermatitis [ 3 ]. 

Essential oils (EOs) are composed of several phytochemicals that protect plants against pathogenic microorganisms. Terpenes and terpenoids are the two main components of EOs classified as the secondary metabolites of various plants. Review of the literature regarding EOs showed that terpenes and terpenoids extracted from different parts of plants contained a wide range of biologically active compounds [ 4 ]. Linalool is an alcoholic terpenoid found in many flowers and spice plants that are extensively used in cosmetic and hygienic industries due to their pleasant odors and definite antimicrobial activities [ 5 - 7 ]. In this regard, previous studies demonstrated that EOs rich in linalool had potent antibacterial and antifungal activities against various microorganisms . Carvone is another terpenoid showing considerable antimicrobial efficacy, as an active component of EOs. 

Due to the lipid-dependency of *Malassezia* species for growth, several methods have been applied for antifungal susceptibility testing (AFST) of these fastidious species [ 11 - 16 ]. Although azole antifungal medications are commonly used to treat *Malassezia* infections, resistant isolates have emerged [ 13 , 17 ]. Therefore, there is interest in the exploration of natural products, like EOs, which contain antimicrobial terpenes and terpenoids, as alternative treatments. Therefore, this study aimed to investigate the antifungal activity of carvone and linalool against *Malassezia* species using a modified urea medium to suggest alternative management for pityriasis versicolor.

## Materials and Methods

### Species Identification

In total, 12 clinical isolates of *Malassezia* species collected from pityriasis versicolor lesions of patients were evaluated in this study. The isolates were cultured on the Leeming Nothman Aagar Medium and were incubated at 32 °C. Genomic DNA was extracted using glass bead and phenol:chloroform:isoamyl alcohol according to the method previously reported [ 18 ].

Amplification of 26S rDNA was performed by a set of primers (*Malf*, 5'-TAACAAGGATTCCCCTAGTA and Malr, 5'-ATTACGCCAGCATCCTAAG). Polymerase chain reaction (PCR) amplification was carried out in a final volume of 50 µl. Each reaction contained 1 µl of DNA template, 0.5 µM of each primer, 0.20 mM of each deoxynucleoside triphosphate, 5 µl of 10× PCR buffer, and 1.25 U of Taq polymerase. An initial denaturation step at 94 °C for 5 min was followed by 30 cycles of denaturation at 94 °C for 45 s, annealing at 55 °C for 45 s, and extension at 72 °C for 1 min, with a final extension step of 72 °C for 7 min. Amplified products were visualized by 1.5% (w/v) agarose gel electrophoresis in Tris/Borate/ Ethylenediaminetetraacetic acid (TBE) buffer, stained with ethidium bromide, and photographed under ultraviolet transillumination. 

The PCR products were digested using *CfoI* restriction enzyme to achieve a species-specific pattern [ 19 ]. Digestion was performed by incubation of 21.5 µl of the PCR product with 10 U of each enzyme in a final reaction volume of 25 µl at 37 °C for 2.5 h, followed by 1.8% agarose gel electrophoresis in TBE buffer and staining with ethidium bromide.

### Media Optimization

Based on the urease activity of *Malasezzia* spp. and previous studies [ 16 , 20 ], Christensen’s urea broth (Merck, USA) was used as the basic medium. The urea broth was supplemented with different concentrations of glycerol, glucose, tween 60, tween 80, and oleic acid to achieve an optimized and transparent medium providing the growth ability for all species. Finally, the pH of the media was adjusted to 6, sterilized by filtration through a 0.22 µm filter, and stored at 4 °C. Under optimal conditions, the color of the medium converted from yellow to pink following the fungal growth.

### Antifungal susceptibility testing

The susceptibility of various species to antifungal medications and monoterpenes was assessed following the guidelines outlined in Clinical and Laboratory Standards Institute document M27-A3. In this study, serial two-fold dilutions of monoterpenes and antifungal agents were prepared in Dimethyl sulfoxide. Range of concentrations for the tested compounds was as follows: 0.15-48 mg/mL of carvone (density of 0.93 g/mL at 25 °C), 0.17-43.2 mg/mL of linalool (density of 0.86 g/mL at 25 °C), 0.001-0.2 mg/mL of amphotericin B (AMB), and 0.003-0.4 mg/mL of ketoconazole (KCZ). All compounds tested were procured from Sigma-Aldrich (St. Louis, MO, USA). Prepared dilutions were then added to 96-well microplates containing 100 µL of enriched broth media. To establish the optimal inoculum for the growth of *Malassezia* species, colonies were suspended in the media and adjusted to an optical density of 1.0 at 530 nm, corresponding to a final inoculum size of approximately 1.0 × 10^3^ to 5.0 × 10^3^ CFU/mL. 

Growth controls (medium with inoculums, but without an antifungal agent) were included. Inoculated microplates were incubated in humid chambers at 32 °C for 4-7 days depending on the species growth rate. MIC was visually defined as the lowest concentration of the antifungal agent that yielded no color conversion. *Candida krusei* (ATCC 6258) and *Cryptococcus neoformans* (H 99) were included in this study as the quality control strains. 

## Results and Discussion

**Figure 1:**
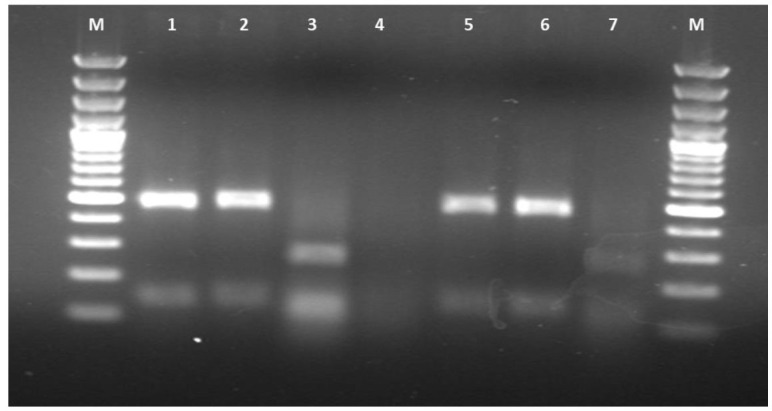
26S rDNA polymerase chain reaction-restriction fragment length polymorphism patterns that digested with *CfoI* restriction enzyme of *Malassezia* clinical strains. Line M: 100 bp DNA ladder; Line 1,2,5,6: *Malassezia globosa*; Line 3,7: *Malassezia furfur*.

All isolated *Malassezia* strains were successfully identified by the amplification of 26S rDNA followed by restriction fragment length polymorphism analyses as *Malassezia globosa* (n=4), *M. furfur* (n=3), *M. slooffiae* (n=3), and *M. sympodialis* (n=2) (Figure 1). 

Given the lipid dependency and positive urease activity of *Malassezia* species, a modified microdilution method based on a colorimetric indicator was introduced. Among different media formulations, Christensen’s urea broth supplemented with tween 60 (0.08%), tween 80 (0.1%), and glycerol (0.2%) yielded a clear medium to make the suitable conditions for the growth of all tested *Malassezia* species (Table 1). 

**Table 1:** Christensen’s urea broth media with different supplementation evaluated for susceptibility testing of *Malassezia* species

Medium	Supplement							Medium quality
	Glucose	Peptone	Tween 40	Tween 60	Tween 80	Glycerol	Oleic acid	
1	5 g	-	0.6%	-	0.1%	-	-	No proper growth of yeasts
2	5 g	-	-	0.08%	0.1%	0.2%	-	Clear/ Optimal growth for all species
3	5 g	1.5 g	-	0.08%	0.1%	0.2%	0.4 %	Turbid
4	5 g	-	-	-	-	-	-	No proper growth of yeasts

Antifungal activities of the antifungal agents as well as the two terpenoids, carvone, and linalool are presented in Table 2. According to the results, AMB resulted in no fungal growth at 0.001-0.003 mg/mL concentrations. In addition, the MICs for KCZ were obtained within the range of 0.006-0.1 mg/mL. Overall, AMB, a fungicide antifungal agent, showed potent *in vitro* antifungal activity against *Malassezia* strains. The tested terpenoids inhibited the growth of the examined *Malassezia* species at 0.3-24 and 0.3-5.4 mg/mL for carvone and linalool, respectively. According to the results, linalool exhibited a higher potent antifungal activity, compared to carvone.

**Table 2:** Minimum inhibitory concentration of carvone, linalool, amphotericin B, and ketoconazole against *Malasezzia* species

No.	Malasezzia species	Carvone (mg/ml)	Linalool (mg/ml)	Amphotericin B (mg/ml)	Ketoconazole (mg/ml)
1	*M. slooffiae*	0.3	1.35	0.001	0.025
2	*M. slooffiae*	0.3	0.68	0.003	0.012
3	*M. slooffiae*	1.5	1.35	0.001	0.100
4	*M. globosa*	24	5.4	0.001	0.025
5	*M. globosa*	6	0.34	0.001	0.025
6	*M. globosa*	12	0.34	0.001	0.006
7	*M. globosa*	24	5.4	0.001	0.050
8	*M. furfur*	24	0.68	0.001	0.012
9	*M. furfur*	12	1.35	0.001	0.025
10	*M. furfur*	24	0.34	0.001	0.012
11	*M. sympodialis*	6	0.68	0.001	0.050
12	*M. sympodialis*	3	0.34	0.001	0.006

Findings of biological evaluations in the present study demonstrated the significant antifungal activity of both monoterpenes against *Malassezia* species. It is worth noting that phenolic and alcoholic natural products are the most active components against fungi [ 4 , 21 ]. As a result, linalool with an alcoholic structure and carvone with a similar structure to phenolic compounds were selected for susceptibility assessment. Linalool, as a cyclic monoterpene containing OH functional group, showed a more effective inhibitory activity against *Malassezia* yeasts, with MICs within the range of 0.3-5.4 mg/mL. The previous studies also emphasized the strong antimicrobial effectiveness of linalool and linalool-rich sources [ 4 , 10 , 22 , 23 ]. In general, compounds with more lipophilicity described as log P (partitioning coefficient of the compounds in octanol/water) have exhibited better efficacy in inhibiting the growth of microorganisms. The lipophilic characteristic of linalool (log P: 2.7) on the one hand and the antimicrobial activity of this compound on the other hand can justify the potential antifungal activity of linalool against these yeasts. These findings supported the previous data that linalool led to the disruption of membrane integrity and blocked the cell cycles of fungal cells [ 5 , 24 ]. Linalool is a “generally recognized as safe” (GRAS) substance approved by the Food and Drug Administration as a direct food additive for humans with an oral lethal dose 50 value of 2,790 mg/kg in rats and a dermal lethal dose 50 value of 5610 mg/kg in rabbits, according to the safety data sheet of Sigma-Aldrich, USA.

Carvone, with a cyclic structure and a ketone functional group, showed antifungal activity against *Malassezia* species, with MICs within the range of 0.3-24 mg/mL. Exact mechanism of action of carvone as an antifungal agent is not fully understood, but several studies have suggested that it may disrupt the fungal cell membrane or inhibit key enzymes involved in the synthesis of the cell wall. Antifungal activity of carvone strongly supports the preceding studies reporting the inhibitory action of carvone through interaction with the cellular components of microorganisms and destabilization of the cell membrane [ 25 ]. However, the structural analysis of carvone with log P: 2.4 and lower lipophilicity represented a lower potency, compared to linalool. Moreover, previous research indicates that carvone exhibits low cytotoxicity on cell lines which supports its safety profile in biological applications [ 26 ]. 

However, there is no standard protocol to determine the susceptibility of *Malassezia* species. Many researchers have developed and suggested different media cultures, inoculum sizes, incubation times, and reading techniques for AFST of *Malassezia* species. Given the differences in lipid requirements of *Malassezia* spp., different media cultures, such as Roswell Park Memorial Institute 1640 and sabouraud dextrose broth were supplemented with various lipid components (tween, glycerol, olive oil, oleic acid, ox bile, or cow milk fat) in order to provide an optimal media for growth and evaluation of the susceptibilities of *Malassezia* spp. in the previous literature [ 13 , 14 , 27 ]. 

Christensen’s urea broth medium is specifically utilized for AFST of *Malassezia* species due to its unique properties that provide the nutritional needs of these yeasts [ 16 , 20 ]. When *Malassezia* species utilize urea, they produce alkaline byproducts that change the pH of the medium, which can be visually monitored using a pH indicator, like phenol red. Additionally, earlier researchers incorporated modifications on urea broth media to assess susceptibility patterns of *Malassezia* spp. that include the general addition of tween 80 and tween 40 [ 16 , 17 ]. In this regard, lipid materials enriched urea broth media to supply fatty acids requirements of *Malassezia* species in accordance with previous reports. As the majority of *Malassezia* species are mostly grown in the presence of tween, enrichment of Christensen’s urea broth with tween 60, tween 80 and glycerol yielded a clear and suitable medium for the growth of these lipophilic species in the present study. Similar to other investigations, results of the current research indicated that the addition of some lipids to the medium, such as oleic acid, led to turbidity as well as difficulties in the colorimetric assessment of the MIC [ 16 ]. 

## Conclusion

Collectively, the preliminary findings on the antifungal activities of carvone and linalool against *Malassezia* yeasts suggest their potential as antifungal therapeutic agents based on natural products. Although Carvone and linalool are recognized as GRAS by the Flavor and Extract Manufacturers Association, their safety and effectiveness as antifungal therapeutic agents need to be evaluated through further rigorous investigations. 
